# The role of sex genotype in paediatric CNS tumour incidence and survival

**DOI:** 10.1007/s00381-021-05165-0

**Published:** 2021-05-05

**Authors:** Wai Cheong Soon, Edward Goacher, Sandeep Solanki, Josie Hayes, Melpo Kapetanstrataki, Susan Picton, Paul Dominic Chumas, Ryan Koshy Mathew

**Affiliations:** 1 Department of Neurosurgery, Queen Elizabeth Hospital Birmingham, Mindelsohn Way, Birmingham, B15 2TH UK; 2 Department of Neurosurgery, Royal Hallamshire Hospital, Glossop Road, Sheffield, England S10 2JF UK; 3 Department of Neurosurgery, University Hospital Coventry and Warwickshire, Clifford Bridge Road, Coventry, CV2 2DX UK; 4 Leeds Genetics Laboratory, St. James’s University Hospital, Leeds Teaching Hospitals NHS Trust, Leeds, LS9 7TF UK; 5 School of Medicine, University of Leeds, Leeds, LS2 9JT UK; 6 Department of Paediatric Neuro-Oncology, Leeds General Infirmary, Leeds Teaching Hospitals NHS Trust, Great George Street, Leeds, England LS1 3EX UK; 7 Department of Neurosurgery, Leeds General Infirmary, Leeds Teaching Hospitals NHS Trust, Great George Street, Leeds, LS1 3EX UK

**Keywords:** Sex, Survival, Incidence, Paediatric, Tumour

## Abstract

**Purpose:**

Evidence exists, in CNS germinomas and medulloblastomas (MB), that patient sex significantly influences incidence and outcome. The role of sex genotype in other paediatric CNS tumours remains unclear. This study sought to examine the role of sex genotype in CNS tumour incidence and overall survival (OS).

**Methods:**

Age-adjusted incidence and OS rates were collected from the Surveillance Epidemiology and End Result (SEER) registry between 2000 and 2011 for common paediatric (<=19 years) CNS tumours: pilocytic astrocytoma (PA), anaplastic astrocytoma, glioblastoma (GBM), medulloblastoma, supratentorial CNS embryonal tumour, ependymoma, and germinoma. All patients with histologically confirmed, ICD-03 coded, first tumours, were included. Kaplan-Meier and Cox regression analyses were used to calculate hazard ratios (HR).

**Results:**

The total cases are as follows: males=3018 and females=2276. Highest incidence was seen in PA (*n*=2103). GBM displayed the worst OS, whilst PA displayed the best. Higher incidence was observed in males for all tumours, except PA. Females with ependymoma had significantly better OS compared to males, whereas males with germinomas had better OS compared to females. Females <1 year with AA had better OS than males. Increasing age significantly improved male and female survival in ependymoma and medulloblastoma.

**Conclusion:**

Interrogating population-based registries such as SEER minimises bias and provides credible data. Observed differences in incidence and OS between the sexes for different paediatric CNS tumours provide useful prognostic information for clinicians. Sex genotype was a significant independent prognostic factor in ependymomas and germinomas. Further investigation of possible epigenetic and hormonal differences may provide sex-specific vulnerabilities that may be exploitable for targeted therapy.

**Supplementary Information:**

The online version contains supplementary material available at 10.1007/s00381-021-05165-0.

## Introduction

Distinct subtypes of central nervous system (CNS) tumours arise in the paediatric population and constitute the second most common childhood malignancy after leukaemia [1]. The exact aetiologies of paediatric CNS tumours remain largely unknown, but genetic predisposition and exposure to ionising radiation have been linked to carcinogenesis, albeit not consistently [2]. Over the last 4 decades, the 5-year relative survival rate has improved from 57 to 74% for paediatric CNS tumours [3]. However, brain cancer is currently the leading cause of cancer death among children and adolescents aged 0 to 19 years [4].

Previous studies in children have been conducted to investigate the influence of differences in sex on cognitive function following surgical and oncological treatment [5–7]. Female patients with brain tumours are at a higher risk of developing more severe neurocognitive deficits when compared to their male counterparts. It has been hypothesised that female patients with brain tumours are more susceptible to white matter damage with adjuvant treatment resulting in lower neurocognitive performance [7]. Sex genotype has been shown to influence the incidence and outcomes of paediatric patients with CNS germinomas and medulloblastomas [8–11]. Despite this, sex is rarely taken into account during the decision-making process when treating patients with CNS tumours. There are several population-based registries such as the US National Cancer Institute’s Surveillance, Epidemiology and End-Results (SEER) Program that report incidence and overall survival (OS) for different paediatric tumours which provide useful prognostic and demographic information for clinicians [3].

Using the SEER registry, this study sought to examine the role of sex genotype in CNS tumour incidence and OS. A clearer understanding of the role of sex-based differences in children with CNS tumour would result in improved prognostication in day-to-day clinical practice and development of tailored sex-specific interventions.

## Methods

### Surveillance Epidemiology and End Results (SEER) data

We interrogated the SEER-18 database for age-adjusted incidence and OS between 2000 and 2011. We looked at common paediatric (<19 years) CNS tumours—pilocytic astrocytoma (PA), anaplastic astrocytoma (AA), glioblastoma (GBM), ependymoma, germinoma, medulloblastoma, and supratentorial CNS embryonal tumours (SCET). Given the retrospective, non-clinical, and open access nature of the SEER-18 database, ethical approval was not sought.

Patients with histologically confirmed, ICD-03 coded, first tumours were included. Year of diagnosis, OS, and mortality status were recorded. Results were analysed by incidence rate, Kaplan-Meier, and Log Rank testing with *p*<0.05 considered to be statistically significant. Multivariable Cox regression was used to generate hazard ratios (HR) and 95% confidence intervals as part of the multivariable analysis for OS. Results were adjusted for year of diagnosis, age, and sex genotype as appropriate. Age cohorts were <1 year (infants), 1–4 years—both together also capture the cohorts in which radiotherapy is generally avoided if possible and then peri-pubertal cohorts. Pre-pubertal was defined as aged 5–9 years for females and aged 5–11 years for males, pubertal was defined as aged 10–14 years for females and 12–16 years for males, and post-pubertal was defined as 15–19 years for females and 17–19 years for males. Statistical analysis was conducted using IBM SPSS version 23.0.

## Results

**Fig. 1:**
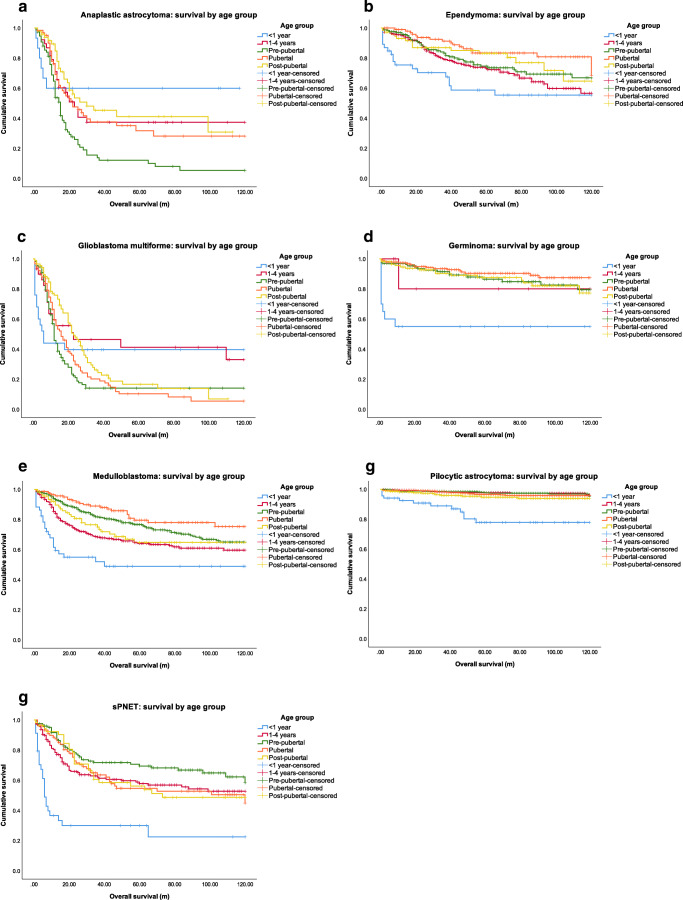
Kaplan-Meier survival curves for age group by tumour subtype. **a** Survival by age group for anaplastic astrocytoma. **b** Survival by age group for ependymoma. **c** Survival by age group for glioblastoma multiforme. **d** Survival by age group for germinoma. **e** Survival by age group for medulloblastoma. **f** Survival by age group for pilocytic astrocytoma. **g** Survival by age group for sPNET. sPENT, supratentorial primitive neuroectodermal tumour

**Fig. 2:**
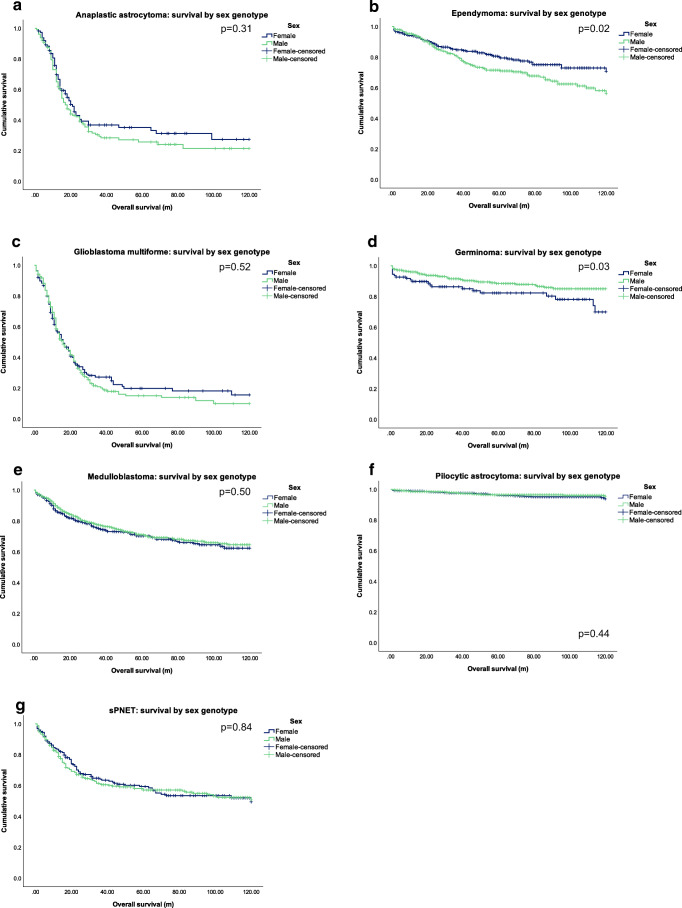
Kaplan-Meier survival curves for sex genotype by tumour subtype. **a** Survival by sex genotype for anaplastic astrocytoma. **b** Survival by sex genotype for ependymoma. **c** Survival by sex genotype for glioblastoma multiforme. **d** Survival by sex genotype for germinoma. **e** Survival by sex genotype medulloblastoma. **f** Survival by sex genotype for pilocytic astrocytoma. **g** Survival by sex genotype for sPNET. sPNET, supratentorial primitive neuroectodermal tumour

In total, 5294 cases were identified and analysed. Of these, 3018 (57.1%) were male. Median age for both males and females were 8 years (range, 0–19 years). Of the 8 tumour subtypes included, PA showed the greatest overall prevalence (39.7%, *n*=2103), followed by MB (19.5%, *n*=1034), and ependymoma (12.3%, *n*=649). A full breakdown of tumour prevalence by sex is shown in Table 1. Over the 10-year period examined, PA consistently displayed the highest incidence when compared to other CNS tumours in the paediatric population. Survival curves for sex genotype by tumour subtype are shown in Fig. 1. Survival curves for age group by tumour subtype are shown in Fig. 2. Appendix 1 compares OS and HRs by sex for each age category.

**Table 1:** Tumour prevalence by sex

Variable	Males *n*=3018 (57.1%)	Females *n*=2276 (42.9%)	Overall *n*=5294
Median age (years) (IQR)	8 (4.14)	8 (3.13)	8 (4.13)
Cancer type, *n* (%)			
Anaplastic astrocytoma	128 (4.2%)	114 (5.0%)	242 (4.6%)
Ependymoma	349 (11.6%)	300 (13.2%)	649 (12.3%)
GBM	207 (6.9%)	137 (6.0%)	344 (6.5%)
Germinoma	355 (11.8%)	109 (4.8%)	464 (8.8%)
Medulloblastoma	660 (21.9%)	374 (16.4%)	1034 (19.5%)
Pilocytic astrocytoma	1063 (35.2%)	1040 (45.7%)	2103 (39.7%)
sPNET	256 (8.5%)	202 (8.9%)	458 (8.7%)

### Anaplastic astrocytoma

Total incidence of AA was 242, of which 52.9% (*n*=128) were male. Median age of diagnosis was 10.0 years for females and 9.5 years for males. Highest incidence was seen in the pubertal age group (*n*=34) for females and the pre-pubertal age group (*n*=53) for males.

No significant difference was seen in median OS between males and female (17.0 months and 21.0 months, respectively, *p*=0.31). Pre-pubertal females showed a significantly increased HR in comparison to females diagnosed with AA aged <1 year (HR 11.6, 95% CI: 1.54–87.3, *p*=0.02). Females showed a significantly increased OS of 19.5 months in comparison to 5.0 months for males (*p*=0.03) in the age category of <1 year.

### Ependymoma

Total incidence of ependymoma was 649, of which 53.8% (*n*=349) were male. Median age at diagnosis was 6.0 years for males and 5.0 years for females. Highest incidence was seen in the 1–4 years age group for both males (52.2%, *n*=133) and females (47.8%, *n*=122).

Females showed a significantly greater median OS in comparison to males (54.5 months vs. 45.0 months, respectively, *p*=0.02). Overall female HR was significantly reduced (HR 0.69, 95% CI: 0.50–0.95, *p*=0.02). Pre-pubertal and pubertal males both showed significantly decreased HRs when compared to males aged <1 year (HR 0.41, 95% CI: 0.20–0.83, *p*=0.01 and HR 0.27, 95% CI: 0.12–0.63, *p*<0.01, respectively). Increasing age showed a significant impact on OS survival for males (HR 0.95, 95% CI: 0.91–0.99, *p*=0.01).

### Glioblastoma

Total incidence of GBM was 344, of which 60.2% (*n*=207) were male. Median age at diagnosis was 12 years for males and 10 years for females. Highest incidence was seen in the pre-pubertal group for both males and female (63.3%, *n*=69 and 36.7%, *n*=40, respectively). Median female OS was 14.0 months, whilst median male OS was 13.0 months (*p*=0.52). There were no significant differences seen in median OS between males and females in any age category.

### Germinoma

Total incidence of germinoma was 464, of which 76.5% (*n*=355) were males. Median age at diagnosis was 11.0 years for females and 14.0 years for males. Highest incidence for females was seen in the pre-pubertal age group (34.3%, *n*=46). Highest male incidence was seen in the pubertal age group (89.9%, *n*=170).

Overall, males showed a significantly greater median OS than females (59.0 months vs. 57.0 months, respectively, *p*=0.03). Overall, females had a significantly higher HR in comparison to males (HR 1.75, 95% CI: 1.03–2.98, *p*=0.04). Both of these findings are heavily influenced by the high significance of increased OS in males aged <1 year and females having a significantly higher HR in comparison to males aged <1 year (HR 8.64, 95% CI: 1.06–70.29, *p*=0.04). For females, increasing age was associated with a significantly decreased HR (HR 0.82, 95% CI: 0.76–0.90, *p*<0.01).

### Medulloblastoma

Total incidence of MB was 1034, of which 63.8% (*n*=660) were male. Median age at diagnosis was 6.5 years for females and 6.0 years for males. Highest incidence for both males and females was seen in the pre-pubertal age group (70.5%, *n*= 306 and 29.5%, *n*=128, respectively).

Median male OS was 42.0 months, in comparison to 39.5 months for females (*p*=0.50). Overall, females displayed a higher HR of 1.08 (95% CI: 0.85–1.38); however, this was not significant (*p*=0.51). All female age groups showed a significantly lower HR (*p*<0.01) in comparison to the age group <1 year (Appendix 1). Only pre-pubertal (HR 0.33, 95% CI: 0.19–0.60, *p*<0.01) and pubertal (HR 0.29, 95% CI: 0.14–0.61, *p*<0.01) males showed a significantly lower HR in comparison to males aged <1 year with MB.

Males showed a significantly greater median OS in the pre-pubertal age group in comparison to females (48.5 months vs. 40.5 months, respectively, *p*=0.02). Females had a significantly higher HR in this age group (HR 1.58, 95% CI: 1.06–2.34, *p*=0.02). Increasing age showed a significantly reduced HR for both males (HR 0.96, 95% CI: 0.93–0.99, *p*<0.01) and females (HR 0.94, 95% CI: 0.91–0.98, *p*<0.01) with MB (Table 2).

**Table 2:** Hazard ratios per additional year of age for individual tumour type

	Overall					Female					Male				
	Unadjusted			Adjusted*		Unadjusted			Adjusted§		Unadjusted			Adjusted§	
Tumour	Median age (range)	HR(95% CI)	*P* value	HR(95% CI)	*P* value	Median age (range)	HR(95% CI)	*P* value	HR(95% CI)	*P* value	Median age (range)	HR(95% CI)	*P* value	HR(95% CI)	*P* value
Anaplastic astrocytoma	10.0 (0–19)	0.99 (0.96–1.01)	0.29	0.98 (0.96–1.01)	0.26	10.0 (0–10)	1.00 (0.96–1.04)	0.91	1.00 (0.96–1.04)	0.97	9.5 (0–19)	0.97 (0.93–1.01)	0.09	0.97 (0.93–1.01)	0.10
Ependymoma	6.0 (0–19)	0.95 (0.93–0.98)	<0.01	0.95 (0.92–0.98)	<0.01	5.0 (0–19)	0.96 (0.91–1.01)	0.07	0.96 (0.91–1.01)	0.08	6.0 (0–19)	0.95 (0.91–0.99)	0.01	0.95 (0.91–0.99)	0.01
Glioblastoma multiforme	11.0 (0–19)	0.99 (0.97–1.02)	0.59	0.99 (0.97–1.01)	0.50	10.0 (0–19)	0.99 (0.95–1.02)	0.41	0.99 (0.95–1.02)	0.43	12.0 (0–19)	1.00 (0.97–1.02)	0.86	0.99 (0.97–1.02)	0.69
Germinoma	13.0 (0–19)	0.94 (0.89–0.99)	0.02	0.95 (0.90–1.00)	0.05	11.0 (0–19)	0.82 (0.75–0.90)	<0.01	0.82 (0.76–0.90)	<0.01	14.0 (0–19)	1.06 (0.98–1.16)	0.17	1.06 (0.97–1.16)	0.17
Medulloblastoma	6.0 (0–19)	0.95 (0.93–0.98)	<0.01	0.95 (0.93–0.97)	<0.01	6.5 (0–19)	0.94 (0.91–0.98)	<001	0.94 (0.91–0.98)	<0.01	6.0 (0–19)	0.96 (0.93–0.99)	<0.01	0.96 (0.93–0.99)	<0.01
Pilocytic astrocytoma	8 (0–19)	0.99 (0.94–1.03)	0.50	0.99 (0.94–1.03)	0.54	8.0 (0–19)	0.93 (0.88–0.99)	0.04	0.93 (0.88–0.99)	0.04	8.0 (0–19)	1.04 (0.98–1.10)	0.23	1.04 (0.98–1.11)	0.22
sPNET	6.0 (0–19)	0.98 (0.95–1.00)	0.06	0.98 (0.95–1.00)	0.07	6.0 (0–19)	0.99 (0.95–1.03)	0.58	0.90 (0.93–1.03)	0.55	6.5 (0–19)	0.93 (0.93–0.99)	0.04	0.96 (0.93–1.00)	0.05

*Adjusted for year of diagnosis and sex genotype

^§^adjusted for year of diagnosis

*HR* hazard ratio, *CI* confidence intervals

### Pilocytic astrocytoma

Total incidence of PA was 2103, of which 50.5% (*n*=1063) were male. Median age at diagnosis was 8.0 years for both males and females. Highest incidence for both males (56.2%, n=379) and females (43.8%, *n*=295) was seen in the pre-pubertal age group.

Median male OS was 65.0 months. Median female OS was 62.0 months (*p*=0.45). All female age groups showed a significantly lower HR (*p*<0.01) in comparison to the age group <1 year (Appendix 1). Males aged 1–4 years (HR 0.11, 95% CI: 0.03–0.42), pre-pubertal (0.16, 95% CI: 0.05–0.53), and pubertal (HR 0.21, 95% CI: 0.06–0.71) all showed significantly lower HRs (*p*<0.01) in comparison to males <1 year. No significant difference in survival between males and females was seen in any age group for PA. Increasing age showed a significant lower HR for females (HR 0.93, 95% CI: 0.88–0.99, *p*=0.04).

### Supratentorial primitive neuroectodermal tumours (sPNET, as per SEER, now more widely recognised as SCET)

Total incidence of SCET was 458, of which 55.9% (*n*=256) were male. Median age at diagnosis was 6.0 years for females and 6.5 years for males. Highest incidence for both males (51.5%, *n*=82) and females (48.5%, *n*=77) was seen in the 1–4 years age group. No significant difference was seen between male (41.0 months) and female (34.5 months) median OS. Pre-pubertal females showed a significantly lower HR in comparison to females aged <1 year with SCET (HR 0.40, 95% CI: 0.16–0.99, *p*<0.05). All male age groups showed significantly lower (*p*<0.01) HRs in comparison to males aged <1 year (Appendix 1).

## Discussion

The overall incidence of childhood cancer is significantly higher in males with an incidence rate ratio of 1.19 [12]. With regard to paediatric CNS tumours, the overall prevalence is higher in males irrespective of tumour subtype, patient age, or region of the world [12, 13]. The intersection of genetic, hormonal, metabolic, and immunological factors in sex differences in CNS tumours has yet to be fully elucidated. Sexual dimorphism in the brain is a recognised phenomenon and has been attributed to gonadal hormone exposure during critical stages of in utero development [14]. Sexual dimorphic mechanisms are thought to influence CNS tumorigenesis and may explain the observed sex disparities in disease prevalence and treatment response. Research has yet to be translated into a sex-specific treatment protocol, which would have the potential to improve decision-making, prognostication, and outcomes.

One of the important differences between male and female epigenetics is the inactivation of the additional X-chromosome in females leading to the formation of Barr bodies [14–16]. The loss or disappearance of the Barr body is observed in female cancer cells and may be accompanied by X-linked gene reactivation [14–16]. The unexplained male predominance across many cancer types has led investigators to believe that a subset of X chromosome (chrX) genes can escape X-inactivation, which in turn could protect females from complete functional loss by single mutation [17]. Dunford et al. found that nearly all of the excess male cancers with ATRX mutations in their dataset were low-grade gliomas [17]. Therefore, they hypothesised that the ATRX gene may escape X-inactivation in certain specific tumour types that may account for the observed sex-bias [17].

Differences in sex hormones that may be responsible in modulating the immune system have been hypothesised to contribute to the observed sex differences in susceptibility to cancer and autoimmune diseases during different stages of life (puberty, reproductive years, post-menopausal)[18–20]. The higher incidence of gliomas in male patients when compared with their premenopausal female counterparts has been attributed to the protective effects of oestrogen. The discovery of oestrogen receptors and aromatase enzymes which converts testosterone to oestradiol in certain gliomas further supports the significant influence of sex hormones in tumour cell proliferation and death [21, 22].

The peak incidence age varies by tumour subtype with the overall peak incidence of paediatric CNS tumours occurring in children under 4 years of age when circulating sex hormones are at their lowest [19, 23]. Evidence in the literature suggests that the sex-based disparities are not due to sex hormones alone but also due to differences in brain size between male and female patients at birth [24], differences in expression of encoded genes on chromosomes responsible for regulating cell growth in male and female embryos [25], and differences in physiology of glucose metabolism in male and female embryos (alteration of glucose metabolism is needed for carcinomatosis, the so-called Warburg effect)[26].

Glioblastoma (GBM) represents one of the most aggressive malignant primary brain tumours [27]. In paediatric GBMs with histone mutation, the male/female incidence is approximately 2:1 when compared with paediatric GBMs without histone mutation or other oncogenic mutations [28]. This highlights the possible role of epigenetic mechanisms in driving tumorigenesis [28]. When looking at the outcome of paediatric patients with GBM in smaller series, sex has not been shown to significantly influence the survival outcomes [29, 30]. Our results complement these findings. McCrea et al. found that female paediatric patients derive a significantly larger survival benefit from gross total resection of high-grade glioma when compared to male patients [31]. In two larger studies of patients with GBM, female patients were noted to have better overall survival [32, 33]. The absence of sex-specific therapy in GBM may be explained by our limited understanding of the underlying hormonal or sex differences in GBM that exists across all stages of life.

Children are ten times more likely to be affected by medulloblastoma than adults [34]. Male to female incidence of medulloblastoma is approximately 1.5 to 1 [34]. However, when the subgroups are examined separately, WNT- and SHH-associated medulloblastomas have been shown to be more prevalent in females, demonstrating an uneven distribution of medulloblastoma subgroups according to sex [35]. Interestingly, Curran et al. found that females greater than 3 years old with medulloblastoma had significantly greater survival than their male counterparts [36]. In our study, we found that sex appears to be a significant factor in the median OS of children with medulloblastoma, but at a different age range and in favour of males (males 48.5 months versus females 40.5 months, pre-pubertal, HR 1.58, 95% CI: 1.06–2.34). At all other ages in our study, there is no significant differences in sex phenotype on survival. There is evidence to suggest that endogenous oestrogens may provide a protective role against medulloblastoma development, possibly via the pro-differentiation and tumour suppressive ligand ERß1 [37, 38]. This mechanism would complement data from Curran et al. [36] suggesting advantageous outcomes for females >3 years of age in comparison to males—suggesting that both sex and age interact to impact outcome in medulloblastoma. Such differences may be explained by the increased difference in oestrogen levels between girls aged >3 years and those <3 years.

Ependymoma represents the third most common brain tumour in the paediatric population, and the OS at 5 years ranges from 24 to 75% [39]. Based on data obtained from the SEER-9 study, the incidence of ependymoma is significantly higher in males [40]. Male outcomes have also been shown to be significantly worse in comparison to females [41]. We found that sex plays a significant role in children diagnosed with ependymoma. After adjusting for year at diagnosis and age, we found that males have a 51% greater risk of death compared to females (*p*=0.013). In a study of 653 children with ependymoma, McGuire et al. found that ependymomas are more common in males [42]. Based on the data drawn from the population-based cancer registry, the authors found that there was a trend towards improved survival among female patients. In another study of 2408 cases of malignant ependymoma, the authors found that the median OS for females with malignant ependymomas was significantly higher compared with males (262 months versus 196 months) [43]. However, the reason as to why female patients survive longer overall is not clear. Differences between sexes are most apparent in subgroups in which epigenetic mechanisms appear to drive tumorigenesis [44].

Intracranial germ cell tumours (GCTs) are rare, typically arising in the pineal or suprasellar regions [45]. They are commonly classified into germinomas and non-germinomatous germ cell tumours. GCTs are significantly more frequently located in the pineal region in males [45]. In addition to differences in location, the histological subtypes of GCT also differ significantly by sex [46]. Despite a distinct variability in gene-specific methylation between histological subtypes of GCT identified by Williams et al., the clear link to sex variability is still yet to be established [47]. Our data analysis has shown that females diagnosed with germinoma have a 1.8 times greater risk of mortality than their male counterparts (*p*=0.037). This is in keeping with the findings of Acharya et al. where the authors found that females with germinomatous germ cell tumours have a higher risk of mortality when compared to their male counterparts [48]. The reason for this is unclear, but we hypothesise that the sex differences in incidence and outcomes of patients with GCTs are due to a combination of DNA methylation variability, epigenetic changes, and brain sexual differentiation from exposure to sex hormones.

As this study has shown, the SEER registry is a useful data source to estimate the cancer burden within a population [49]. However, the quality of the SEER data is heavily reliant on the accuracy of the pathological reports [49]. Its main limitation is that it does not currently publish individual brain tumour histopathological results and does not provide integrated molecular results. This information is important due to the heterogeneity of brain tumours, the natural history, and the response to treatment between tumours with the same phenotypes but with different genotypes, all of which could influence incidence and OS [50].

## Conclusion

Sex genotype was a significant independent prognostic factor in overall survival in both ependymomas and germinomas. In medulloblastoma and ependymoma, increasing age appears to be a protective factor. Registries such as SEER can deliver useful and substantial data for clinicians to better comprehend the cancer burden within a population. It is imperative that SEER publishes the specific integrated tumour histopathological results as there are numerous different molecular subtypes of CNS tumours. This may reveal further relationships between sex genotype and outcomes from CNS tumours, enabling further research into the complex molecular, hormonal, epigenetic, and developmental interactions in paediatric CNS tumours. Understanding the sex-based differences in incidence and OS in children with CNS tumours is a fundamental step towards improving prognostication and developing more effective targeted therapy.

## Supplementary information

ESM 1 (DOCX 27 kb)

## Data Availability

Freely available on the SEER registry.
