# Supplementary material for: The role of sex genotype in paediatric CNS tumour incidence and survival
Source: Childs Nerv Syst. 2021 May 5;37(7):2177–86. doi: 10.1007/s00381-021-05165-0 (PMC8263540; doi:10.1007/s00381-021-05165-0)
Supplement: Supplementary file 1 — (DOCX 27 kb) [file 381_2021_5165_MOESM1_ESM.docx]

| **Tumour** | **Incidence (F:M)** | **Female 1-year survival** | **Male 1-year survival** | **Female 5-year survival** | **Male 5-year survival** | **Female Median survival (m)** | **Male Median survival (m)** | **P** | **Female HR (95% CI)*** | **P** |
| --- | --- | --- | --- | --- | --- | --- | --- | --- | --- | --- |
| Anaplastic Astrocytoma  Total  <1  1-4  Pre-pubertal^§^  Pubertal^§^  Post-pubertal^§^ | 114:128  8:7  21:17  23:53  34:30  28:21 | 65.8%  75.0%  76.2%  52.2%  61.8%  71.4% | 71.1%  25.0%  76.5%  66.0%  73.3%  90.4% | 15.8%  25.0%  19.0%  8.7%  14.7%  17.9% | 14.1%  28.6%  29.4%  7.5%  13.3%  14.3% | 21.0  19.5  25.0  12.0  22.0  26.0 | 17.0  5.0  23.0  15.0  25.0  34.0 | 0.31  0.03  0.71  0.49  0.97  0.78 | 0.85 (0.61 – 1.16)  0.07 (0.01 – 1.01)  1.15 (0.49 – 2.71)  1.21 (0.70 – 2.10)  1.02 (0.54 – 1.93)  0.98 (0.44 – 2.19) | 0.30  0.05  0.75  0.50  0.95  0.96 |
| Ependymoma  Total  <1  1-4  Pre-pubertal^§^  Pubertal^§^  Post-pubertal^§^ | 300:349  22:24  122:133  67:102  46:60  43:30 | 84.3%  59.1%  81.1%  85.1%  95.6%  93.0% | 87.4%  75.0%  88.0%  90.2%  88.3%  83.3% | 46.7%  54.5%  41.8%  44.8%  47.8%  58.1% | 40.4%  25.0%  35.3%  42.2%  60.0%  53.3% | 54.5  67.5  47.5  53.0  57.5  65.0 | 45.0  37.5  35.0  49.0  56.5  61.0 | 0.02  0.42  0.09  0.68  0.53  0.11 | 0.69 (0.50 – 0.95)  0.69 (0.26 – 1.80)  0.63 (0.38 – 1.03)  0.87 (0.45 – 1.67)  0.76 (0.28 – 2.08)  0.44 (0.15 – 1.23) | 0.02  0.45  0.06  0.68  0.59  0.12 |
| Glioblastoma Multiforme  Total  <1  1-4  Pre-pubertal^§^  Pubertal^§^  Post-pubertal^§^ | 137:207  11:14  14:16  40:69  35:57  37:51 | 55.5%  36.4%  42.9%  40.0%  65.7%  73.0% | 10.2%  50.0%  56.3%  58.0%  57.9%  62.7% | 58.5%  9.1%  35.7%  5.0%  11.4%  5.4% | 7.2%  14.3%  12.5%  2.9%  3.5%  11.8% | 14.0  3.0  9.5  9.0  16.0  22.0 | 13.0  12.0  15.0  12.0  13.0  20.0 | 0.52  0.60  0.80  0.46  0.10  0.67 | 0.92 (0.72 – 1.19)  1.61 (0.57 – 4.53)  1.11 (0.40 – 3.10)  1.19 (0.76 – 1.85)  0.67 (0.42 – 1.09)  1.12 (0.67 – 1.86) | 0.53  0.37  0.84  0.44  0.11  0.66 |
| Germinoma  Total  <1  1-4  Pre-pubertal^§^  Pubertal^§^  Post-pubertal^§^ | 109:355  11:9  3:4  46:88  19:170  30:84 | 86.2%  27.2%  66.7%  93.5%  100.0%  93.3% | 87.9%  77.8%  75.0%  86.4%  88.2%  90.5% | 47.7%  9.1%  33.3%  54.3%  42.1%  56.7% | 49.6%  66.7%  50.0%  37.5%  54.7%  50.0% | 57.0  2.0  11.0  63.0  47.0  76.0 | 59.0  68.0  44.5  42.0  65.0  59.5 | 0.03  <0.01  0.22  0.80  0.59  0.83 | 1.75 (1.03 – 2.98)  8.64 (1.06 – 70.29)  105.97 (0.01 – 1.7^E+12^)  1.09 (0.41 – 2.91)  0.61 (0.08 – 4.66)  0.83 (0.27 – 2.59) | 0.04  0.04  0.70  0.86  0.64  0.75 |
| Medulloblastoma  Total  <1  1-4  Pre-pubertal^§^  Pubertal^§^  Post-pubertal^§^ | 374:660  19:32  118:195  128:306  60:83  49:44 | 80.5%  47.4%  73.7%  93.8%  90.0%  83.7% | 82.9%  62.5%  81.5%  85.9%  86.7%  75.0% | 36.4%  10.5%  34.7%  39.1%  43.3%  34.7% | 37.6%  21.9%  33.8%  42.8%  36.1%  31.8% | 39.5  11.0  37.5  40.5  54.0  39.0 | 42.0  19.5  36.0  48.5  45.0  37.0 | 0.50  0.47  0.57  0.02  0.49  0.21 | 1.08 (0.85 – 1.38)  1.48 (0.65 – 3.35)  0.89 (0.59 – 1.34)  1.58 (1.06 – 2.34)  0.72 (0.32 – 1.65)  0.61 (0.28 – 1.32) | 0.51  0.35  0.57  0.02  0.44  0.21 |
| Pilocytic Astrocytoma  Total  <1  1-4  Pre-pubertal^§^  Pubertal^§^  Post-pubertal^§^ | 1040:1063  37:30  267:296  295:379  275:248  166:110 | 91.2%  83.8%  92.1%  91.5%  92.0%  89.2% | 90.0%  83.3%  91.9%  89.4%  90.7%  87.3% | 52.9%  48.6%  52.4%  53.9%  53.8%  51.2% | 54.5%  29.6%  57.1%  54.5%  55.2%  54.5% | 62.0  58.0  61.0  63.0  65.0  61.0 | 65.0  50.5  71.0  65.0  66.5  65.0 | 0.45  0.44  0.27  0.77  0.87  0.33 | 1.21 (0.76 – 1.93)  1.59 (0.45 – 5.62)  1.89 (0.61 – 5.78)  1.17 (0.45 – 3.04)  0.92 (0.34 – 2.45)  0.61 (0.20 – 1.81) | 0.43  0.48  0.27  0.74  0.87  0.37 |
| sPNET  Total  <1  1-4  Pre-pubertal^§^  Pubertal^§^  Post-pubertal^§^ | 202:256  13:21  77:82  42:81  35:54  35:18 | 77.2%  41.2%  71.4%  81.0%  82.9%  91.4% | 78.1%  23.8%  78.0%  86.4%  85.2%  83.3% | 36.6%  23.1%  33.8%  42.9%  25.7%  51.4% | 43.0%  14.3%  43.9%  53.1%  40.7%  33.3% | 34.5  6.0  31.0  45.0  32.0  61.0 | 41.0  6.0  43.0  65.0  42.0  34.0 | 0.84  0.14  0.77  0.65  0.34  0.32 | 0.97 (0.73 – 1.29)  0.54 (0.22 – 1.32)  0.92 (0.56 – 1.49)  1.15 (0.60 – 2.22)  1.34 (0.71 – 2.53)  0.68 (0.30 – 1.51) | 0.81  0.18  0.72  0.67  0.37  0.34 |

*adjusted for year of diagnosis. ^§^pre-pubertal defined as aged 5-9 for females and aged 5-11 for males, pubertal defined as aged 10-14 for females and 12-16 for males, post-pubertal defined as 15-19 for females and 17-19 for males.
